# Wrist-ankle acupuncture (WAA) for primary dysmenorrhea (PD) of young females: study protocol for a randomized controlled trial

**DOI:** 10.1186/s12906-017-1923-9

**Published:** 2017-08-22

**Authors:** Yingfan Chen, Sinan Tian, Jing Tian, Shi Shu

**Affiliations:** 1 Changhai Hospital of Traditional Chinese Medicine, Second Military Medical University, 168 Changhai Road, Yangpu District, Shanghai, China; 2 Department of Nursing Science, Second Military Medical University, Shanghai, China

**Keywords:** Wrist-ankle acupuncture (WAA), Primary dysmenorrheal (PD), Randomized controlled trial

## Abstract

**Background:**

Primary dysmenorrhea (PD) is one of the most common health complaints all over the world, specifically among young females. Acupuncture has been employed to relieve the pain-based symptoms and to avoid the side effects of conventional medication, and wrist-ankle acupuncture (WAA) has confirmed analgesic efficacy for various types of pain. The aim of this study is to evaluate the immediate analgesia effect of WAA on PD of young females.

**Methods/design:**

This study will carry out a randomized parallel controlled single-blind trial to observe the immediate analgesia effect of WAA in PD of young females. Sixty participants who meet inclusion criteria will be recruited from September 2016 to September 2017 in Changhai hospital of China. They are randomly assigned to WAA therapy or sham acupuncture groups (30 patients for each group), and then receive real or sham acupuncture treatment, respectively. In this trial, the primary outcome measure is simple form of McGill pain questionnaire (SF-MPQ), while expectation and treatment credibility scale (ETCS), safety assessment, the COX menstrual symptom scale (CMSS), questionnaire about the feeling of being punctured are included in the secondary outcomes.

**Discussion:**

This trial will be the first study protocol designed to evaluate the immediate analgesia effect of WAA in PD of young females. The strengths in methodology, including rigorous randomized, sham-controlled, participants-blinded and assessors-blinded, will guarantee the quality of this study. WAA doesn’t require any needling sensation, so non-penetrating sham acupuncture can serve as an effective placebo intervention in this trial.

**Trials registration:**

Chinese Clinical Trial Registry (identifier: ChiCTR-IOR-16008546; registration date: 27 May 2016).

## Background

Dysmenorrhea is a common disease in females of reproductive age [1], leading to the activity limitation, efficiency impairment, and even life quality decrease. Moreover, the vast majority is primary dysmenorrhea (PD), defined as painful menses in women with normal pelvic anatomy. Due to the lack of standard methods for assessing the severity of dysmenorrhea, studies based on the different definitions of the condition have reported the prevalence of PD as between 45% and 95% of menstruating women [2]. PD has negative impact on physical and psychological aspects of health, and its harm to the health will in turn aggravate the symptoms of PD, which is a vicious cycle. It has become an important research field to alleviate the symptom of PD safely and to improve the quality of life and well-being rating of female.

Therapeutically, the conventional pharmacotherapy for the PD is the use of non-steroidal anti-inflammatory drugs (NSAIDs) and oral contraceptives [3, 4]. Although NSAIDs and other drugs can alleviate the pain of PD significantly, their application is limited, mostly because of their disability in curing the disease ultimately, along with their side effects and unaffordable high prices [5, 6].

Professor Zhang Xinshu and his colleagues from Changhai Hospital of Second Military Medical University, Shanghai, China invented and developed wrist-ankle acupuncture (WAA), a modern subcutaneous acupuncture technique, in the 1970s [7]. WAA which does not produce any pain or “needling sensation” is quite distinguished from the traditional acupuncture [8]. According to its unique theoretical system and the clinical evidence, WAA has demonstrated analgesic effects for both chronic and acute pain [9–11]. In addition, the active principle is considered to have correlation with the threshold of pain, or the regulation function of central nervous system, which is partly consistent with the analgesic mechanism of the traditional acupuncture [12–16]. There is a guaranteed guiding significance of WAA for PD’s treatment. To date, there are few studies investigating the application of WAA in the alleviation of PD. The present study is aimed at investigating the immediate analgesia effects of WAA for young females with PD.

## Methods/design

### Objectives

The aims of this study are to:

1. observe the immediate analgesia effect of WAA in PD of young females;
2. provide high-quality evidence-based recommendations on further treatment.

### Hypothesis

WAA therapy would relieve the pain of patients more effectively than sham acupuncture.

### Study design

**Fig. 1:**
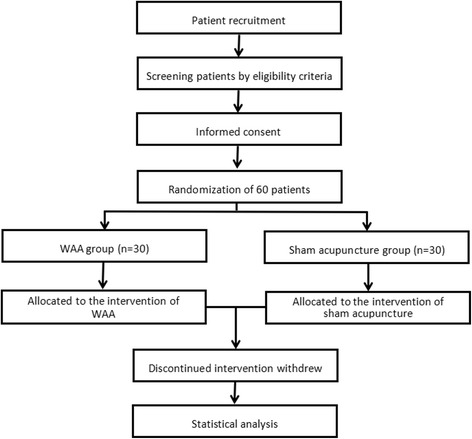
Flowchart of the study design

The randomized parallel controlled single-blind trial was designed to observe the immediate analgesia effect of wrist-ankle acupuncture in primary dysmenorrhea of young females (Fig. 1). The study will be conducted in Changhai hospital of China from September 2016 to September 2017.

### Participants and recruitment

Posters of this trial will be put on notice boards to recruit potential participants in the hospital. And the trial information leaflets will be distributed to the patients. The protocol details are explained to the participants in the leaflets and posts. Participants will be included only if they meet the inclusion criteria and provide written informed consent.

### Key inclusion and exclusion criteria

#### Inclusion criteria

1. patients conformed to the diagnostic criterion of PD according to the Primary Dysmenorrhea Consensus Guidelines [17];
2. nulliparous women of 18 to 30 years old;
3. instant pre-treatment VAS score of over 40 mm;
4. no experience with treatment of WAA;
5. signature of informed consent form.

#### Exclusion criteria

1. secondary dysmenorrhea caused by endometriosis, uterine myoma, endometrial polyps, pelvic inflammatory disease, or other gynecological problems confirmed by a gynecological abdominal ultrasound B examination;
2. females with irregular/infrequent menstrual cycles (outside of the typical range 21 to 35 days’ cycle);
3. patients complicated with severe diseases (e.g. cerebral, liver, kidney, or hematopoietic system diseases), or mental defects;
4. take analgesic during the past 12 h;
5. received other alternative therapies such as massage, acupressure, herbal therapy in the previous three months;
6. poor treatment compliance (e.g. unstable working and living situation, difficult follow-up).

### Data collection

Two identical scale questionnaires should be completed before and after the treatment for assessing the pain-based symptoms of PD in the current menstrual cycle. Participants will complete the first questionnaire 10 min before the treatment, while the second one will be collected immediately after the treatment. The researchers will be present when the questionnaires are completed to ensure the reliability and validity.

### Interventions

**Fig. 2:**
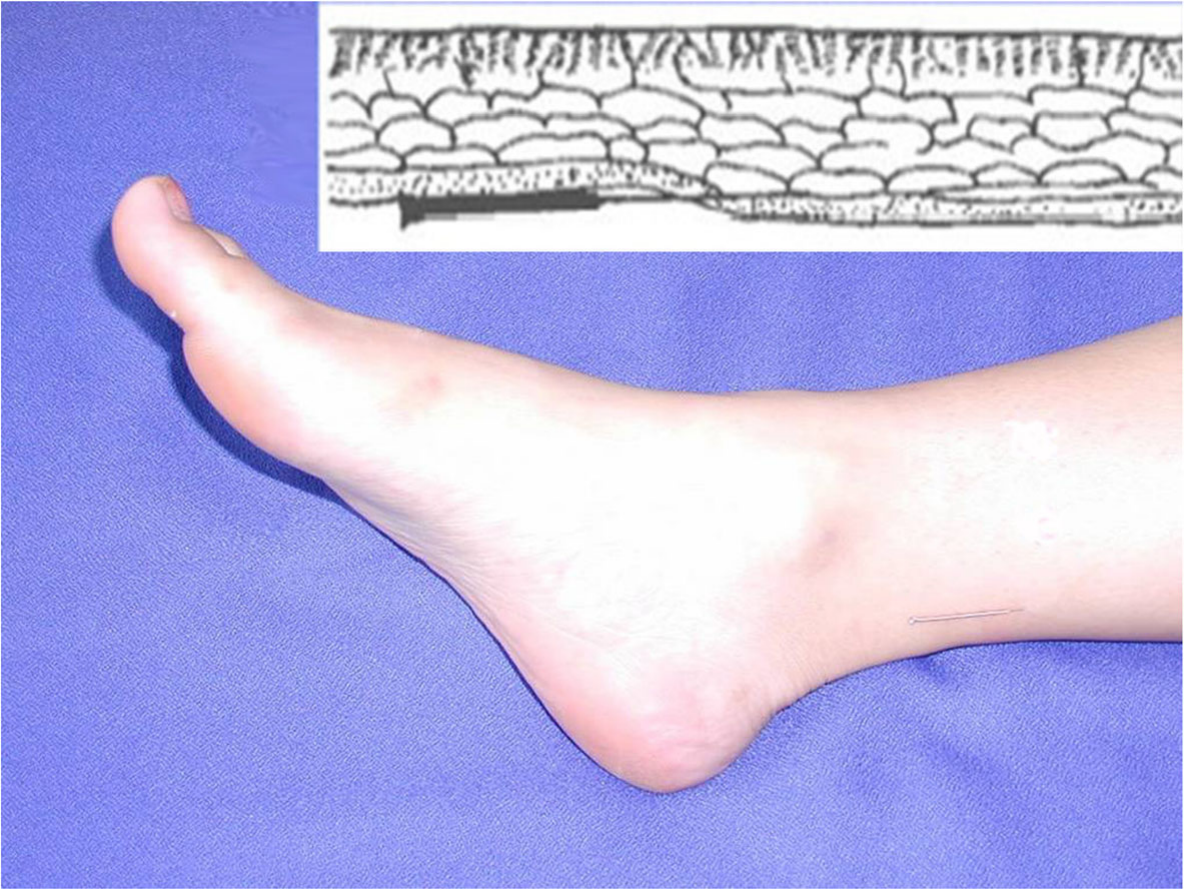
WAA needling point 1 on the ankle zone (Point lower 1) after insertion. The location of Point lower 1 is close to the medial border of tendo calcaneus

According to the theory of WAA, each side of the body and each limb are longitudinally divided into six zones and one needling point is defined in each zone at the wrist or ankle. Needling the certain point will relieve the pain on the corresponding zone of the point. If the pain is on the upper (lower) part of the body (with the diaphragm as the demarcation), the needling point at the ipsilateral wrist (ankle) is selected [7, 18]. The pain sites of PD patients in the study are primarily located in the lower abdomen. To standardize the selection of the needling site and the treatment protocol, point 1 at both ankles will be needled (Fig. 2). Participants will be randomly assigned to the WAA group or the sham acupuncture group. The intervention will be applied during the first 24 h of the most intense dysmenorrhea’s occurrence. A single registered acupuncturist, with at least one year of previous WAA experience, will administer the care to all subjects.

#### WAA group

WAA was administered on point 1 at both ankles on the first day when the pain occurs during the period. Retain the needle for 30 min. The subjects were asked to wear an eye mask. A disposable sterile WAA needle (0.25 mm in diameter and 25 mm in length, Suzhou Medical Appliance Factory, Jiangsu Province, China) was chosen in this trial. The target point was disinfected with an iodophordis infectant (Shanghai Likang Disinfectant Hi-tech Co. Ltd.). The processed needles were also held with thumb, index finger, and middle finger of the right hand. The skin near the target point was gently pressed with the left thumb to make it slightly taut. Then, the needle tip was swiftly inserted into the skin at the target point at an angle of 30°. The needle was lowered to the horizontal position and slowly advanced until the entire needle (except the handle) entered the subcutaneous tissue. The handle was then fixed to the skin with an adhesive tape. The needles were retained in the subcutaneous tissue for 30 min. Upon withdrawal of the needle, dry sterilized cotton balls will be firmly applied to the insertion points. The patient will only feel a negligible stabbing pain when the tip of the needle pierces the skin.

#### Sham acupuncture group

**Fig. 3:**
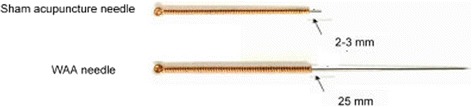
WAA needle & Sham acupuncture needle. WAA needle is the disposable sterile needle (0.25 mm in diameter and 25 mm in length, Suzhou Medical Appliance Factory, Jiangsu Province, China), sham acupuncture needle is also used the same model needle (0.25 mm in diameter and 25 mm in length, Suzhou Medical Appliance Factory, Jiangsu Province, China), while the tip and most body was cut off and blunted with only 2-3 mm of the needle body remained

Sham acupuncture was administered on point 1 at both ankles on the first day when the pain occurs during the period. Retain the needle for 30 min. The subjects were asked to wear an eye mask. The target site was also disinfected with an iodophordis infectant (Shanghai Likang Disinfectant Hi-tech Co. Ltd.). The tip and most body of the disposable sterile acupuncture needle will be cut off and blunted (0.25 mm in diameter and 25 mm in length, Suzhou Medical Appliance Factory, Jiangsu Province, China) (Fig. 3). This sham needle has been successfully used in the previous trail design [19]. Only 2-3 mm of the needle body in length was remained. The processed needles were also held with three right-hand fingers (thumb, index finger, and middle finger). The skin near the target site was gently pressed with the left thumb to make it slightly taut. The needle tip was swiftly punctured the skin at the target point at an angle of 30° (The tip actually was not inserted into the skin). Then, the needles remained in the point of skin horizontally for 30 min. The handle was also fixed to the skin with an adhesive tape. For a successful sham treatment, the patient will also only feel a little negligible stabbing pain when the tip of the needle pierces the skin, and no other needling sensation.

### Randomization and blinding

An outside researcher who is not allowed to directly contact with the participants will perform computerized randomization. The assessor will also be blinded to the treatment allocation. The acupuncturist performing the WAA intervention and sham WAA intervention can’t be completely blinded, but he is not allowed to reveal any information about treatment procedures and outcomes to the participants or the assessor. Thus, both the participants and the assessor won’t distinguish clearly which intervention was given. A sealed envelope containing allocation sequence number for each participant will be opened after each participant is confirmed to meet the eligibility criteria and informed consent is made. If any error or disclosure with regard to randomization occurs, a new randomization sequence will be generated starting from the problematic serial number and applied to the participant from then on.

### Outcome measures

#### The primary outcome

Simple form of McGill pain questionnaire (SF-MPQ):

The SF-MPQ consists of 3 parts, including the Visual Analogue Scales (VAS), Present Pain Intensity scale (PPI) and Pain Rate Index (PRI). The main component is composed of 15 descriptors, including 11 sensory and 4 affective, and the descriptors are rated on an intensity scale (0 = none, 1 = mild, 2 = moderate or 3 = severe).

#### Secondary outcomes

The COX menstrual symptom scale (CMSS) will be rated as the main secondary outcomes. The CMSS consists of 18 items and each item has 5 grades according to symptom severity, including the evaluation on general frequency of menstrual symptoms and average severity. 0 point for absence of discomfort; 1 point for mild discomfort; 2 points for moderate discomfort; 3 points for severe discomfort; and 4 points for extremely severe.

Expectation and treatment credibility scale (ETCS), safety assessment, participants’ feeling of acupuncture questionnaire will also be included.

#### Sample size calculation

The sample size was determined according to the results of our previous pilot study, a two-arm design with WAA group and sham acupuncture group (the response rate was approximately 80% and 40%, respectively, showed by the pilot study). On the basis of the calculation performed by PASS11 with a two-sided significance level of 0.05 and power of 0.80, a total sample size of 52 would be required, with 26 for each group. Considering a maximum dropout tolerance of 15%, 30 initial participants in each group are required for this trial.

### Statistical analysis

SPSS15.0 statistical software packages will be used to analyze the data. The statistician is blinded from the allocation of groups. For quantitative data, the distribution pattern and homogeneity of variance will be examined. If there is a symmetric distribution, the mean (M) ± standard deviation will be used for statistical description. Both paired samples t-test between the quantitative indices before and after treatment in one group and independent t-tests between the two groups will be used to determine and compare the effect of acupuncture. The entire statistical test will use bilateral examination, and the significance level sets at *P* < 0.05.

## Discussion

The result of this trial is expected to provide high-quality clinical evidence that WAA is effective for young females with PD. Acupuncture has been used worldwide for various types of pain [20, 21], and the evidence from clinical studies also suggested that WAA has confirmed analgesic efficacy for many kinds of pain, like post-TACE pain or some cancer pain [15, 21–23]. Nowadays, the incidence of PD is increasing, especially among young females. At present, there are few attentions paid to the application of WAA for PD. Therefore, a trial about this direction deserves our devotion.

Based on TCM acupuncture theory, filiform needle therapy on the acupionts always needs participants to get the sensation of obtaining qi, which is usually experienced as sourness, numberness, distention and pain. Only the acupuncture trial design with request of obtaining qi can reflect the correct and obvious therapeutic effect of acupuncture [24]. Nevertheless, patients who experienced acupuncture treatment will perceive the difference between non-penetrating sham acupuncture and real acupuncture easily. For this reason, it is hard for common filiform needle in acupuncture intervention designed as a blind trial. However, Wrist-Ankle Acupuncture doesn’t require any needling sensation. In the trial, the subjects were asked to wear an eye mask, so it is hard for them to distinguish the certain intervention in the following treatment. For this reason, we believe that non- penetrating sham acupuncture can serve as an effective placebo intervention in this trial. Meanwhile, participants’ feeling of acupuncture questionnaire would arrange in the end of treatment in order to exclude breaking blind subjects. Therefore, a randomized controlled single-blind trial with a non- penetrating sham wrist-ankle acupuncture group was designed, and it will be able to evaluate the immediate analgesia effect of WAA in PD of young females and provide useful advice of evidence-based medicine for further treatment.

### Trial status

The trial is currently recruiting patients.

## Abbreviations

CMSS: COX menstrual symptom scale
PD: Primary Dysmenorrhea
SF-MPQ: Simple form of McGill pain questionnaire
TACE: Transcatheter arterial chemoembolization
WAA: Wrist-Ankle Acupuncture

## References

[1] Kennedy S (1997). Primary dysmenorrhoea. Lancet.

[2] Proctor M, Farquhar C (2006). Diagnosis and management of dysmenorrhea. BMJ.

[3] French L (2005). Dysmenorrhea. Am Fam Physician.

[4] Dawood MY (2006). Primary dysmenorrheal: advances in pathogenesis and management. Obstet Gynecol.

[5] Nevatte T, O’Brien PM, Bäckström T, Brown C, Dennerstein L, Endicott J, Epperson CN, Eriksson E, Freeman EW, Halbreich U, Ismail K (2013). ISPMD consensus on the management of pre-menstrual disorders. Arch Women’s Ment Health.

[6] Zhang WY, Li WPA (1998). Efficacy of minor analgesics in primary dysmenorrhoea: a systematic review. BJOG Int J Obstet Gynaecol.

[7] Zhang XS, Ling CQ, Zhou QH (2002). Practical wrist-ankle acupuncture therapy.

[8] Lao HH. Wrist-ankle acupuncture: methods and applications. 2nd ed. New York: Oriental Healthcare Center 1999: 1–43.

[9] Zhu ZZ, Wang XP (1998). Clinical observation on the therapeutic effects of wrist-ankle acupuncture in treatment of pain of various origins. J Tradit Chin Med.

[10] Su JT, Zhou QH, Li R (2010). Immediate analgesic effect of wrist-ankle acupuncture for acute lumbago: a randomized controlled trial. Chinese Acupuncture & Moxibustion.

[11] Marra C, Pozzi I, Ceppi L (2011). Wrist-ankle acupuncture as perineal pain relief after mediolateral episiotomy: a pilot study. Journal of Alternative & Complementary Medicine.

[12] Yang J, Huang J (2007). Electro-acupuncture analgesia can enhance the counteraction to pain via the p38 MAPK signal pathway. Nervous Diseases and Mental Health.

[13] Wang ZF, Yu XM, Tao J (2016). Study on the analgesic effect of wrist ankle acupuncture and its application in Fmri. Chinese Journal of Ethnomedicine and Ethnopharmacy.

[14] Zhou YL, Liu YJ, Fu JN, Wei W (2017). Effect of Huaisanzhen on central analgesic transmitters in the rat of the nerve root pain caused by protrusion of lumbar intervertebra disc. Chinese Acupuncture & Moxibustion.

[15] Li BZ, Chan WC, Lo KC (2014). Wrist-ankle acupuncture for the treatment of pain symptoms: a systematic review and meta-analysis. Evidence-Based Complementray and Alternative Medicine.

[16] Xia HU, Wei GU, Zhou QH (2005). Analgesic efficacy and mechanism of wrist-ankle acupuncture on pain caused by liver cancer. Chinese Journal of Integrated Traditional & Western Medicine on Liver Diseases.

[17] Lefebvre G, Pinsonneault O, Antao V, Black A, Burnett M. Feldman K: Primary dysmenorrhea consensus guideline. J Obstet Gynaecol Can. 2005,27(12):1117–1146. 10.1016/s1701-2163(16)30395-4 16524531

[18] Zeng K, Dong HJ, Chen HY (2014). Wrist-ankle acupuncture for pain after transcatheter arterial chemoembolization in patients with liver cancer: a randomized controlled trial. Am J Chin Med.

[19] Shu S, Zhan M, You YL (2015). Wrist-ankle acupuncture (WAA) for precompetition nervous syndrome: study protocol for a randomized controlled trial. Trials.

[20] Gemma M, Nicelli E, Gioia L (2015). Acupuncture accelerates recovery after general anesthesia: a prospective randomized controlled trial. Journal of Integrative Medicine.

[21] Lin JG, Chen YH (2012). The role of acupuncture in cancer supportive care. Am J Chin Med.

[22] Hu X, Ling CQ (2004). Biomechnical mechanism of analgesic effect of wrist-ankle acupuncture. Chinese Acupuncture & Moxibustion.

[23] Hu X, Ling CQ, Zhou QH (2004). Clinical observation on wrist-ankle acupuncture for treatment of pain of middle-late liver cancer. Chinese Acupuncture & Moxibustion.

[24] To M, Alexander C (2015). The effects of park sham needles: a pilot study. Journal of Integrative Medicine.

